# Translation, validation and extended factor models of the German State Difficulties in Emotion Regulation Scale (S-DERS)

**DOI:** 10.1186/s40479-025-00299-y

**Published:** 2025-07-01

**Authors:** M. Sicorello, M. Elsaesser, D. R. Kolar

**Affiliations:** 1 Department of Psychosomatic Medicine and Psychotherapy, Central Institute of Mental Health, Medical Faculty Mannheim, Heidelberg University, Heidelberg, Germany; 2 German Center for Mental Health (DZPG), Partner Site Mannheim, Mannheim, Germany; 3 Department of Psychiatry and Psychotherapy, Medical Center– University of Freiburg, Faculty of Medicine, University of Freiburg, Freiburg, Germany; 4 Department of Psychology, University of Regensburg, Regensburg, Germany

**Keywords:** Emotion regulation, Psychometrics, Psychopathology, Assessment, Structural equation models

## Abstract

**Background:**

Difficulties in emotion regulation are a key transdiagnostic factor in mental health disorders. While much research has focused on emotion regulation difficulties as stable, trait-like constructs, emotion regulation is inherently dynamic, unfolding over time. This highlights the need for state-like measures to capture these temporal dynamics in both laboratory and real-world contexts, such as the State Difficulties in Emotion Regulation Scale (S-DERS). The present study aimed to (a) translate the S-DERS into German, (b) validate its psychometric properties, and (c) provide novel examinations whether state-based emotion regulation difficulties share an underlying general factor or are interconnected but distinct sub-components, complemented with an exploratory network approach.

**Methods:**

A sample of 214 participants, predominantly young females, completed the 21-item German version of the S-DERS following a negative mood induction procedure. Participants also completed a broader battery of psychological assessments. Factor structure, reliability, and construct validity were examined using confirmatory factor analysis and exploratory structural equation modeling (ESEM) with cross-loadings. These were compared to bi-factor, higher-order, and network models.

**Results:**

The German S-DERS demonstrated a robust four-factor structure, high reliability, and strong construct validity, consistent with the original English version. ESEM indicated that extensive cross-loadings were necessary to achieve good model fit. A four-factor correlated model outperformed both bifactor and higher-order models, suggesting that emotion regulation difficulties are best conceptualized as four distinct but interrelated constructs without a shared general factor: (a) Non-acceptance of Current Emotions, (b) Limited Ability to Modulate Current Emotional and Behavioral Responses, (c) Lack of Awareness of Current Emotions, and (d) Lack of Clarity about Current Emotions.

**Conclusions:**

The German version of the S-DERS is a reliable and valid tool for assessing state-like difficulties in emotion regulation. The extended factor models highlight the multidimensional nature of emotion regulation difficulties, with complex interrelations among distinct but related constructs. These insights can inform future research on emotion dysregulation and support efforts to validate the S-DERS in clinical populations.

**Supplementary Information:**

The online version contains supplementary material available at 10.1186/s40479-025-00299-y.

## Introduction

Emotion regulation is a key concept for mental and physical well-being, comprising all implicit and explicit strategies affecting the occurrence, intensity, or duration of emotional experiences [16]. There are many stages at which these processes can fail, starting from the identification of emotions, over the selection of a strategy, its implementation, and the monitoring of success [9]. One particularly influential clinical model outlined six categories of emotion regulation difficulties, which can negatively affect a person’s well-being [13]: A *Lack of Awareness* as the diminished tendency to attend and acknowledge one’s emotions, a *Lack of Clarity* as the extent to which people understand their emotional experiences, the *Non-Acceptance* of primary emotions in the form of secondary meta-emotional responses (e.g., shame or anger for feeling a certain way), a limited access to helpful *Strategies*, difficulties engaging in *Goal-Directed Behavior* as well as *Impulsive Behavior *when confronted with negative emotions. This model gave rise to the six factor Difficulties in Emotion Regulation Scale (DERS), which has been used in thousands of empirical studies, repeatedly demonstrating the relevance of these dimensions for the etiology and maintenance of psychopathology across diagnostic boundaries [5, 6, 28, 39].

Originally, most emotion regulation studies focused on trait-like constructs assessed with cross-sectional questionnaires—including the DERS—at a single time point, primarily measuring a person’s general concept of their own emotional processes. Still, emotions and their regulation typically unfold over brief time periods [15]. Studies in the laboratory or daily life can address this state-like dynamic nature of emotion regulation difficulties as they emerge, which is the fundamental building block of trait-like difficulties [9]. Therefore, Lavender and colleagues [22] used original items of the 36-item DERS to construct the 21-item State Difficulties in Emotion Regulation Scale (S-DERS) for momentary assessments. Based on an experimental induction of negative mood and exploratory factor analysis, they determined that state difficulties are best captured by a smaller item set comprising four factors instead of the original six DERS factors. This solution merged the factors for strategies, goal-directed behavior, and impulsiveness into a single factor reflecting the limited ability to *Modulate *current emotional and behavioral responses. It has been applied to a large range of clinical topics such as eating disorders, alcohol use, sleep disruption, and daily affective experiences [7, 11, 33, 36].

In the present study, we aimed to advance research on state-like emotion regulation difficulties by (a) providing a translated and validated German version of the S-DERS and (b) testing competing structural models to determine whether state-based difficulties reflect a single overarching construct or distinct but interrelated subcomponents. For the translation and validation, we aimed to perform a conceptual replication of the original S-DERS construction study, which included an experimental induction of negative mood to provide an appropriate affective context for emotion regulation difficulties to occur and was conducted in a sample of young women. We preregistered hypotheses that (H1) our mood induction increased negative affect based on the valence dimension, (H2) we replicate the original four-factor structure, (H3) internal consistency reliability will be satisfactory (> 0.80) for all scales except for the Clarity scale, which had limited internal consistency in the original study (> 0.60), (H4) there is convergent validity between the S-DERS and the trait-DERS scales, and (H5) concurrent criterion validity for other relevant constructs from the clinical and personality domain. Specifically, we expected positive associations of the global S-DERS score with psychopathology, neuroticism, and affective reactivity to the mood induction. Concerning S-DERS subscales, we expected positive associations between Non-Acceptance and experiential avoidance, Modulate and impulsivity, Awareness and mindfulness, as well as Clarity and mindfulness.

Concerning the extended factor models, even for the trait-DERS, it is still unclear whether there exists a common latent construct of emotion regulation difficulties or distinct but correlated components [1, 38, 42]. This question is of theoretical relevance, but also crucial to decide whether the total score or subscale scores should be preferred in a given research application. The original four-factor solution of the S-DERS was based on exploratory factor analysis (EFA) with parallel analysis. While this provided initial support for the scale’s structure, theory-driven confirmatory modeling approaches are needed to determine whether state-based difficulties in emotion regulation entail a single overarching construct (similar to the p-factor of psychopathology) or distinct but interconnected domains.

**Fig. 1:**
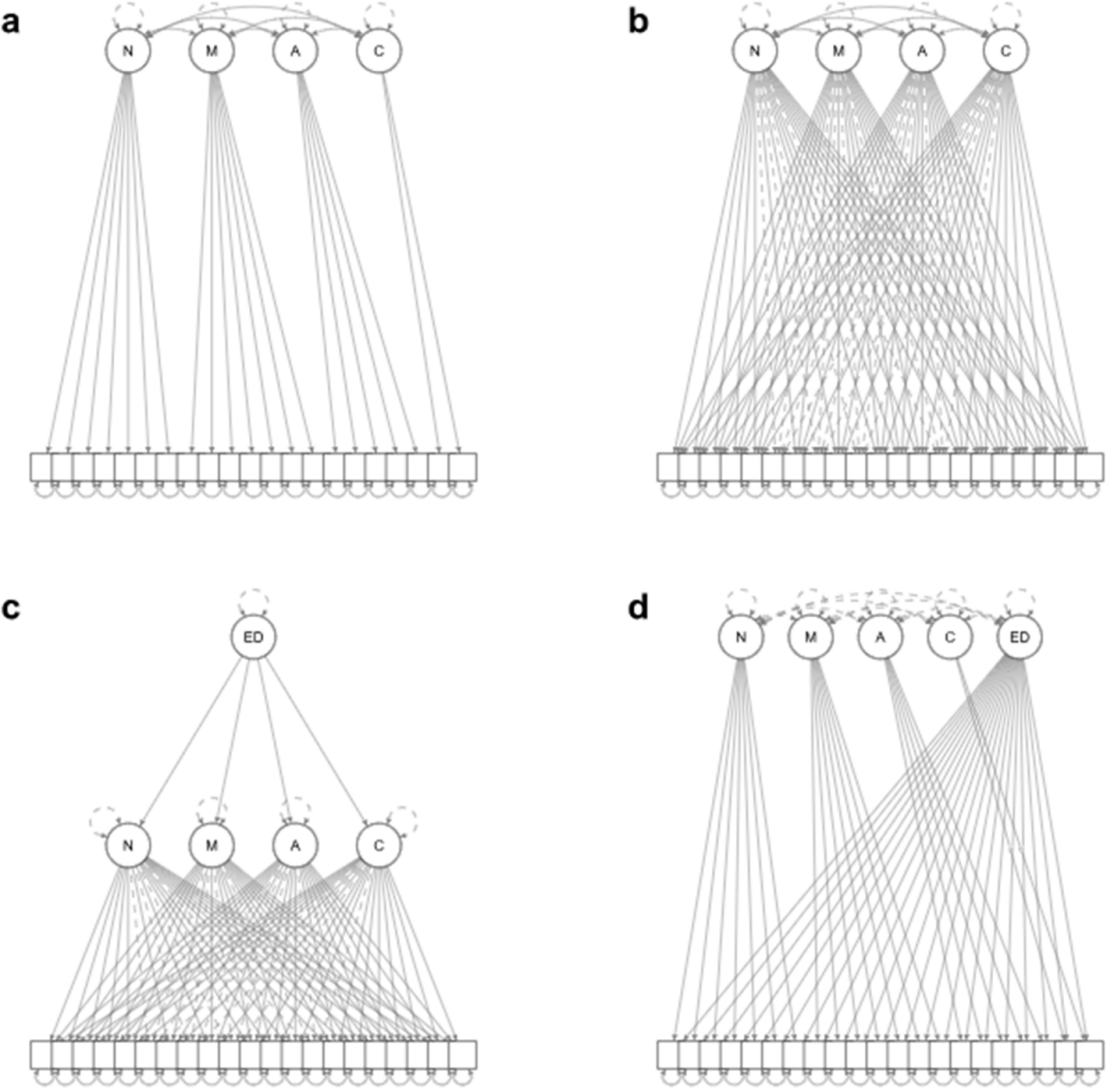
Factor models tested for the S-DERS. **a** CFA simple structure without cross-loadings. **b** ESEM with cross-loadings. **c** Model with a higher-order factor. **d** Bi-factor model. ED = Emotion regulation difficulties. N = Non-Acceptance. M = Modulate. A = Awareness. C = Clarity

To address this question, we started by fitting a simple structure confirmatory factor analysis without a general factor, where each indicator only loads on a single scale and no general factor is assumed (Fig. 1a). Still, even for the trait-DERS models without cross-loadings fit measures are often insufficient [17]. Exploratory structural equation modeling (ESEM) provides a framework to fit a model with cross-loadings for all items and has provided improved model fit for the trait DERS in the past as well as a reduction in factor correlations [17, 24, 38]. These models entail the perspective that observed behaviors, like indicated questionnaire responses, are complex and multidetermined. Therefore, we also conducted an ESEM-based analysis with fit measures as the success criterion, which in terms of strictness is between inference based on CFA simple structures and EFA loading sizes (Fig. 1b).

Next, we computed a higher-order and a bifactor model to test for a superordinate general factor of state-based emotion regulation difficulties. Conceptually, a higher-order model implies that a general latent construct of emotion regulation difficulties causally influences the four subscales, which in turn causally influence the participant responses to the items (Fig. 1c). As a competing non-preregistered alternative, the bifactor model implies a direct effect of global difficulties on indicators, which is distinct from the specific constructs underlying the subscale factors (Fig. 1d). Further, as the existence of cross-loadings might indicate more complex relations between indicators, we provide an exploratory network analysis as a hypothesis-generating tool to support future research on the causal structure of emotion regulation difficulties [4].

## Methods

### Participants and procedures

The sample comprised 214 participants of ages 18–61, which were predominantly young (mean age = 22.5, *SD* = 6.5) female (89%, 10% male, one person gender-diverse) university students (93%). This composition is overall similar to the original S-DERS validation study, which only included young adult women between the age of 18–25. In our sample, 51 participants (24%) reported a diagnosis for a mental disorder in the past, with 26 (12%) having received treatment.

Following the literature on power simulations for confirmatory factor analysis, we aimed to recruit at least 200 participants [44]. An a priori stopping rule was set if recruiting plateaus after 200 participants (< 8 participants per week for 4 weeks in a row) or reaches a maximal sample size of 250. According to power calculations with G*Power (v3.1) for one-tailed zero-order correlations, a sample size of 200 allows a statistical power of 90% to detect a population correlation of 0.20 or higher. This can be viewed as sufficient, given the moderate-to-high correlations needed to attest validity. Custom-simulations using the simsem package showed that a simple structure CFA based on the factor structure of Lavender and colleagues (2017) is unbiased for our sample size using the criteria of Wolf and colleagues (2013; i.e., relative bias in coefficients and standard errors below 0.05 and confidence interval coverage of the population parameter in at least 90% of 10,000 iterations). These criteria were also fulfilled for a more complex bi-factor model, simulated from a population model with general- and specific-factor loadings of 0.50, except that standard errors are slightly underestimated, albeit to a degree which maintained confidence interval coverage above the acceptable threshold (median relative standard error bias = 0.06, range −0.12–0.01) Participants were recruited over mailing lists, social media, and internal advertisements at Heidelberg and Regensburg University. As a participation incentive, 25 online coupons á 40€ were provided after study completion to randomly drawn participants. The only inclusion criterion was age between 18–65. The study was performed contactless on participants’ smartphones using the SEMA3 platform [32]. This included a demographic section, followed by a section with psychological questionnaires, a mood induction, and the translated S-DERS. This procedure took on average 31.4 min (*SD* = 20.6). Psychology students could participate for course credit, which was taken by 88% of participants.

### Translation

The S-DERS (21 items) was separately translated into German by M.S., M.E., and D.K. without reference to the German DERS version. Then, translations were compared for consistency and discussed, followed by a comparison to the German DERS version to resolve conflicts, settling on a consensual translation. The final German items were retranslated to English by two independent psychologists with no significant differences between the back-translation and the original English S-DERS items.

The translated items can be found in Table S1 (ordered by factor) and Table S2 (administration order with English items for comparison). Item responses ranged from 1 (“Not at all”/“Überhaupt nicht”) to 5 (“Completely”/”Völlig”). The items of the Awareness scale were reversed and then sum scores for the total scale and the subscales calculated. Notably, while this scoring is consistent with the previous procedure, it differently weighs the four subscales, as they consist of a different number of items. We present exploratory analyses on the differences between this and an unweighted procedure.

### Further psychological measures

Other psychological measures used for validation included the trait-DERS (36 items, [17]), the Neuroticism scale of the NEO Five Factor Inventory (12 items, [3]), the Depression, Anxiety, and Stress Scale (DASS-21, 21 items, [31]), the Barrett Impulsivity Scale (BIS-15, 15 items, [26]), the Acceptance and Action Questionnaire II (AAQ-II, 7 items, [19]), and Awareness and Describe Subscales of the Five Facet Mindfulness Questionnaire Short (FFMQ, 16 items, [27]). Lastly, the Interpersonal Emotion Regulation Questionnaire (20 items; [35] and the Childhood Trauma Questionnaire [21]) were administered for a different research project and are therefore not reported in the present study.

### Mood induction

Before the assessment of the S-DERS, all participants underwent a negative mood induction, as negative emotions are likely a prerequisite for the meaningful assessment of momentary emotion regulation difficulties [22]. The mood induction consisted of a combination of sad music and autobiographical recall [43]. Participants listened to 6 min of the ‘Adagio for Strings’ by Samuel Barber while writing about a recent negative experience. This negative experience was instructed to be a 5–6 on a scale from 0 (not a negative experience) to 10 (extremely negative experience). Immediately before and after the mood induction, participants indicated their momentary affective state based on a two-dimensional affective grid with the dimensions *valence* (from 0 = unpleasant emotion to 10 = pleasant emotion) and *arousal *(from 0 = low energy to 10 = high energy; [40]. Lastly, as another manipulation check, participants were asked how well they could immerse themselves in the recalled negative experience (from 1 = not at all to 5 = very well).

### Data analysis

The success of the mood induction was tested as the difference between affective valence before versus after the manipulation using a paired t-test. An exploratory test on the affective arousal dimension is also reported without previously specified expectations.

**Fig. 2:**
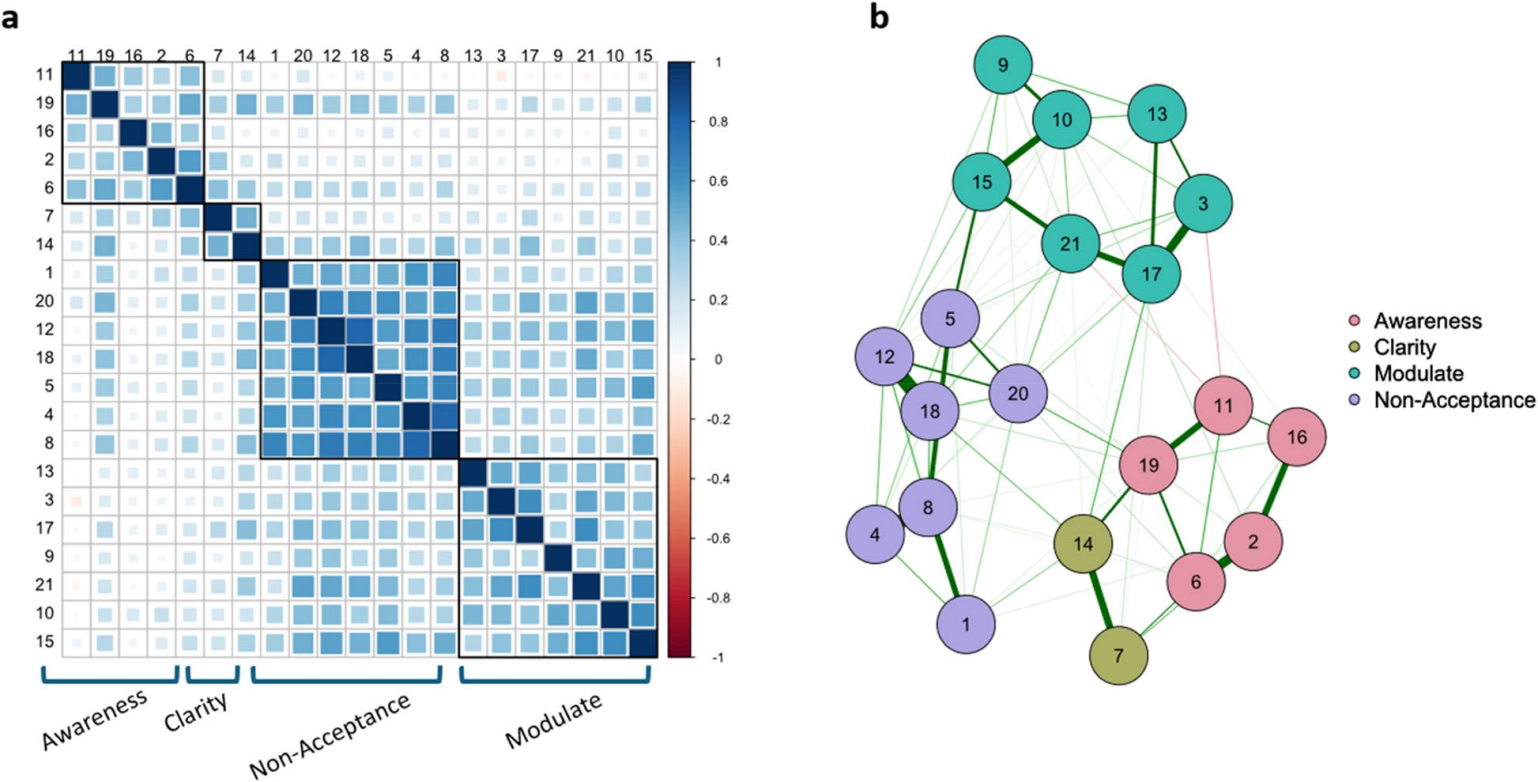
**a **Correlations between S-DERS items. Clusters were determined using hierarchical agglomerative clustering with complete linkage. **b** Network analysis based on the partial correlation matrix

All factor models tested for the S-DERS are shown in Fig. 1. First, we conducted a confirmatory factor analysis for four factors without cross-loadings (i.e., simple structure; Fig. 1a) using maximum likelihood estimation with the R package Lavaan [37]. We preregistered that if model fit measures did not meet the criteria of RMSEA ≤ 0.06; CFI ≥ 0.95; TLI ≥ 0.95; SRMR ≤ 0.08 [20], we test an ESEM model with cross-loadings for all items (Fig. 1b) using the esem package [34]. In this procedure, first, an EFA was fit with the psych package using principal axis factoring and promax rotation, which was also used in the original S-DERS validation. Referent items were identified using the automated procedure of the esem package. Then, this model was refit as a CFA in Lavaan with the loadings of the referent items fixed to their EFA loadings, while the remaining EFA loadings were used as starting values. If this model also did not meet the fit measure criteria above, we preregistered to perform EFA with the number of factors based on parallel analysis at the 99% quantile, with loadings > 0.40 considered as substantial loadings on the main factors and loadings > 0.30 considered as substantial cross-loadings. This EFA procedure is identical to that used in the original validation study. Internal consistency (composite reliability) was calculated as McDonald’s ⍵ from the final model [10]. Additionally, the higher-order (Fig. 1c) and bifactor models (Fig. 1d) were tested against the winning model using the Vuong test [41]. Lastly, we conducted an exploratory network analysis on the partial correlation matrix (Fig. 2). Pruning of edges was performed based on the Extended Bayesian Information Criterion with λ = 0.5 and graphical lasso using the bootNet package [8].

For associations between the S-DERS and other variables, bivariate correlations were computed with a preregistered one-tailed α = 0.05. All analyses were performed in R (v4.3.2).

### Open science practices

Preregistration, codebook, data, and analysis code can be found at: https://osf.io/ebj6u/. For reproducibility or other reuse of data and code, we recommend directly accessing the github repository, which also includes a documentation of files, version control, and steps to reproduce analyses: https://github.com/MaurizioSicorello/SDERSvalid_Analysis. A print version of the questionnaire can be found in both repositories as well as the supplements.

There were two notable changes to the preregistration. First, we originally preregistered to conduct EFA with Geomin rotation and maximum likelihood estimation. This is in contrast to the analyses performed by Lavender et al. [22] and was considered as the esem package did not exist at the time of preregistration and the only validated procedure was performed with this estimation procedure. Using the same procedure as Lavender et al. [22] is an improvement not only due to consistency, but also because principal axis factoring is better suited to identify factors with few items, such as the Clarity scale which only consists of two items. Secondly, we preregistered likelihood ratio tests for model comparisons, but the net procedure indicated models resulting from CFA and ESEM were not nested. Possibly, this was due to the selective fixing of values in ESEM, thus making the Vuong test for non-nested models the safer option [41]. Both procedures led to the same inferential decision.

## Results

### Manipulation check (H1)

Affective valence decreased after the mood induction, indicating a successful manipulation, albeit with a small-to-moderate effect size (*t*(213) = 4.58, *p* < 0.001, *d*_c_ = 0.31; *M*_pre_ = 5.9*, SD*_pre_ = 1.9; *M*_post_ = 5.3*, SD*_post_ = 2.1). Exploratory analyses indicated that affective arousal also decreased with a slightly larger effect size (*t*(213) = 5.9, *p* < 0.001, *d*_c_ = 0.40; *M*_pre_ = 5.6*, SD*_pre_ = 1.9; *M*_post_ = 4.9*, SD*_post_= 1.9), further confirming the successful induction of sadness, which is generally classified as a low arousal emotion [2]. Overall, most participants stated they were successfully immersed in the experience (*M* = 3.8, *SD* = 0.9).

### Basic factor structure and extended higher-order models (H2)

The correlations between items visually indicated a solution with four factors and considerable cross-loadings (Fig. 2a). For the four-factor simple structure CFA without cross-loadings, none of the fit measures crossed the preregistered threshold (Table 1). The four-factor ESEM structure with cross-loadings improved model fit significantly, while only SRMR crossed the threshold. This model also outperformed the higher-order and bifactor models (Table 1) and no modification indices were statistically significant after Bonferroni-Holm correction. A parallel analysis also indicated a four-factor solution. Overall, the loading pattern conformed to the original S-DERS validation except for two cross-loadings (Table 2): Item 17 of the Modulate scale (“My emotions feel out of control”) had a substantial cross-loading of 0.46 on the Clarity factor; item 7 of the Clarity scale had a substantial cross-loading of 0.37 on the Awareness factor (see Discussion).

**Table 1:** Fit measures and inferential statistics for competing structural equation models

	SRMR	RMSEA	CFI	TLI	AIC	BIC	Ӽ^2^	*df*	Model Comparison	*p*-Value
CFA Simple Structure	0.078	0.094	0.850	0.828	10,849	11,011	527.6	183		
ESEM Cross-loadings	0.039	0.078	0.923	0.881	10,729	11,049	313.3	136	Model 2 > Model 1	≥ 0.001
ESEM Higher-order	0.076	0.086	0.904	0.854	10,770	11,083	357.9	138	Model 2 > Model 3	≥ 0.001
Bi-factor	0.066	0.082	0.893	0.867	10,764	10,973	414.3	169	Model 2 > Model 4	0.008

Based on *N* = 214 participants

*SRMR *standardized root mean square residual, *RMSEA* root mean square error of approximation, *CFI* comparative fit index, *TLI* Tucker–Lewis index,* CFA *confirmatory factor analysis, *ESEM* exploratory structural equation model, *EFA *exploratory factor analysis, *AIC* Akaike information criterion, *BIC *Bayesian information criterion

**Table 2:** Factor loadings and correlations

	Factor			
Item	1	2	3	4
Factor 1: Non-acceptance of Current Emotions				
Item 8	**0.97 (0.91)**	−0.12 (0.48)	0.00 (0.29)	0.03 (0.38)
Item 4	**0.91 (0.82)**	−0.07 (0.43)	−0.06 (0.2)	−0.06 (0.28)
Item 12	**0.71 (0.83)**	0.19 (0.62)	−0.06 (0.23)	0.04 (0.41)
Item 18	**0.69 (0.78)**	0.05 (0.53)	−0.01 (0.26)	0.17 (0.47)
Item 1	**0.68 (0.68)**	−0.08 (0.37)	0.06 (0.27)	0.06 (0.33)
Item 5	**0.59 (0.73)**	0.29 (0.61)	0.06 (0.28)	−0.13 (0.26)
Item 20	**0.53 (0.72)**	0.24 (0.6)	0.09 (0.33)	0.05 (0.4)
Factor 2: Limited Ability to Modulate Current Emotional and Behavioral Responses				
Item 10	−0.06 (0.42)	**0.87 (0.77)**	0.14 (0.26)	−0.22 (0.16)
Item 21	−0.02 (0.47)	**0.71 (0.77)**	−0.09 (0.11)	0.21 (0.48)
Item 9	0.02 (0.35)	**0.64 (0.59)**	0.05 (0.16)	−0.15 (0.14)
Item 3	−0.03 (0.38)	**0.62 (0.66)**	−0.15 (0.03)	0.21 (0.43)
Item 13	−0.12 (0.31)	**0.61 (0.6)**	−0.03 (0.11)	0.17 (0.37)
Item 15	0.23 (0.58)	**0.6 (0.72)**	0.07 (0.25)	−0.1 (0.27)
Item 17	−0.08 (0.42)	**0.54 (0.67)**	−0.07 (0.14)	**0.46 (0.64)**
Factor 3: Lack of Awareness of Current Emotions				
Item 6 (r)	0.03 (0.32)	0.00 (0.24)	**0.70 (0.75)**	0.16 (0.35)
Item 2 (r)	−0.09 (0.17)	0.06 (0.16)	**0.67 (0.66)**	0.02 (0.18)
Item 11 (r)	−0.04 (0.09)	−0.09 (0.01)	**0.62 (0.58)**	−0.02 (0.08)
Item 16 (r)	−0.13 (0.09)	0.13 (0.13)	**0.61 (0.56)**	−0.11 (0.04)
Item 19 (r)	0.25 (0.46)	−0.09 (0.27)	**0.54 (0.66)**	0.22 (0.42)
Factor 4: Lack of Clarity about Current Emotions				
Item 14	0.14 (0.44)	−0.03 (0.37)	0.13 (0.34)	**0.66 (0.74)**
Item 7	−0.16 (0.17)	0.04 (0.22)	**0.37 (0.44)**	**0.47 (0.51)**

Structure coefficients are in parentheses. Bold coefficients mark absolute loadings larger than. 30. (r) indicated reversed items. The structure of this table is based on Lavender et al. [22] for ease of comparison

The four factors were moderately to highly correlated, with the lowest correlation between Modulate and Awareness (*r* = 0.18) and the largest between Modulate and Non-Acceptance (*r* = 0.58) the latter being visible as a large block in Fig. 2a. Notably, this correlation was even higher in the validation of the original S-DERS. In-between were the correlations for Non-Acceptance and Awareness (*r* = 0.30), Non-Acceptance and Clarity (*r* = 0.43), Awareness and Clarity (*r* = 0.35), and Modulate and Clarity (*r* = 0.41). Overall, this pattern of factor correlations resembled those of the original S-DERS validation, except for larger correlations between the Awareness scale and the remaining three scales.

In contrast, the network model based on partial correlations emphasizes the proximity of the Awareness and Clarity scales (Fig. 2b). Also, it indicates that items 5 (“I am feeling very bad about myself”) and 15 (“I believe that I am going to end up feeling very depressed”) might serve as connectors between the Non-Acceptance and the Modulate clusters (see Discussion).

### Reliability (H3)

As expected, reliabilities were sufficient for the total scale and all subscales except the Clarity scale, converging with the pattern of the original validation study (in parentheses): Total Score: ⍵ = 0.94 (0.86), Non-Acceptance: ⍵ = 0.92 (0.92), Modulate: ⍵ = 0.79 (0.85), Clarity: ⍵ = 0.63 (0.65).

### Construct and criterion validity (H4-H5)

The S-DERS subscales all had the highest correlations with their analogue trait-DERS subscales (Table 3). Still, notably, almost all subscales from both questionnaires had substantial intercorrelations. Together with ESEM-like structure of the four factors with considerable cross-loadings, this indicates a moderate specificity of subscales, inherent to all English and German versions of the trait and state DERS [13, 17, 22]. A similar pattern emerged for the remaining external constructs: Experiential avoidance most strongly correlated with Non-Acceptance, but also very strongly with the Modulate subscale. Mindful Description appeared to be more specific to the Awareness subscale, as hypothesized, while Mindful Acting with Awareness was strongly correlated to all subscales, with the strongest correlation for the Modulate scale.

**Table 3:** Correlations between S-DERS scales and other constructs

	S-DERS				
	Total	Non-Acceptance	Modulate	Awareness	Clarity
DERS	**0.78**	**0.64**	**0.63**	**0.55**	**0.48**
DERS: Nonacceptance	**0.64**	**0.69**	**0.44**	**0.32**	**0.30**
DERS: Awareness	**0.44**	**0.27**	**0.16**	**0.68**	**0.35**
DERS: Clarity	**0.47**	**0.31**	**0.24**	**0.53**	**0.55**
DERS: Strategies	**0.75**	**0.63**	**0.68**	**0.40**	**0.39**
DERS: Impulse	**0.58**	**0.42**	**0.57**	**0.32**	**0.36**
DERS: Goals	**0.46**	**0.36**	**0.50**	**0.20**	**0.19**
DASS-21	**0.58**	**0.45**	**0.60**	**0.30**	**0.29**
Neuroticism	**0.58**	**0.46**	**0.57**	**0.33**	**0.24**
Affective Reactivity: Valence	−0.12	−0.07	−0.15	−0.08	−0.07
AAQ-II	**0.64**	**0.51**	**0.58**	**0.37**	**0.39**
BIS	0.10	0.01	**0.12**	0.08	**0.15**
FFMQ: Describe	**−0.29**	**−0.14**	**−0.12**	**−0.48**	**−0.26**
FFMQ: Awareness	**−0.44**	**−0.27**	**−0.46**	**−0.29**	**−0.33**

*(S-)DERS *(State) Difficulties in Emotion Regulation Scale, *DASS-21 *Depression, Anxiety, and Stress Scale, *BIS-15 *Barrett Impulsivity Scale, *AAQ-II* Acceptance and Action Questionnaire II, *FFMQ *Five Facet Mindfulness Questionnaire (short form)

Statistically significant correlations (one-sided, α =.05) are shown in bold. The cutoffs for one-sided tests at different common statistical thresholds were:

*p* <.05 (one-sided): 0.11

*p* <.01 (one-sided): 0.16

*p* <.001 (one-sided): 0.21

There were two surprising findings, which deviated from our expectations. First, the correlations between the BIS-15 scale for impulsivity and the S-DERS subscales were much smaller than expected, albeit still statistically significant for the preregistered association with the Modulate scale, as well as the non-preregistered association with the Clarity scale. Post-hoc analyses indicated this is because only the BIS-15 subscale for attentional impulsivity was substantially related to the S-DERS scales. For this subscale, the correlations conformed to the expectations, with the strongest effect for the Modulate scale at *r* = 0.32 (Non-Acceptance: *r* = 0.16, Awareness: *r* = 0.12, Clarity: *r* = 0.23).

The second surprising finding was the absence of the expected correlation between affective reactivity to the mood induction and the global S-DERS. In fact, the association went in the opposite direction, with higher state emotion regulation difficulties showing a small association with a stronger decrease in negative affect. These reactivity scores were calculated from two variables: affective valence before minus after the mood induction. Therefore, we inspected the correlations with the two separate variables (pre and post measurement). This showed that the global S-DERS score was substantially correlated with lower (i.e., more negative) affect before but not after the mood induction (before: *r* = −0.22, *p* = 0.001; after: *r* = −0.07, *p* = 0.260). To test whether this observation is specific to the S-DERS, we repeated these correlation analyses with the neuroticism scores instead of S-DERS scores as a trait-based construct for negative affective reactivity, showing the same pattern (reactivity: *r* = −0.18, *p* = 0.008; before: *r* = −0.28, *p* < 0.001; after: *r* = −0.08, *p* = 0.231). In sum, both the S-DERS and neuroticism were more strongly correlated to affect before than after the mood induction, albeit with the plausible effect direction of more negative affect (smaller values) being associated with more emotion regulation difficulties and neuroticism (higher values). To understand how two correlations in a psychologically plausible direction can lead to an unexpected effect direction for a difference score, consider the covariance decomposition:

$$\begin{array}{ll}&\mathrm{cov}(\mathrm S\text{-}\mathrm{DERS},\;\mathrm{Reactivity})\\=&\mathrm{cov}(\mathrm S\text{-}\mathrm{DERS},\;{\mathrm{Affect}}_{\mathrm{pre}}\;-\;{\mathrm{Affect}}_{\mathrm{post}})\\=&\mathrm{cov}(\mathrm S\text{-}\mathrm{DERS},\;{\mathrm{Affect}}_{\mathrm{pre}})\;-\;\mathrm{cov}(\mathrm S\text{-}\mathrm{DERS},\;{\mathrm{Affect}}_{post})\end{array}$$

Inserting different correlation sizes in the last line shows, if the covariance between the S-DERS and affect *before* the manipulation is more negative than after the manipulation, the observed effect will have a negative sign (i.e., the “wrong” direction). Consequently, if the covariance between the S-DERS and affect *after* the manipulation is more negative than before the manipulation, the observed effect will have a positive sign (i.e., the “right” direction). We will expand on potential psychological reasons in the Discussion section.

### Weighted versus unweighted subscales (Post-hoc)

**Fig. 3:**
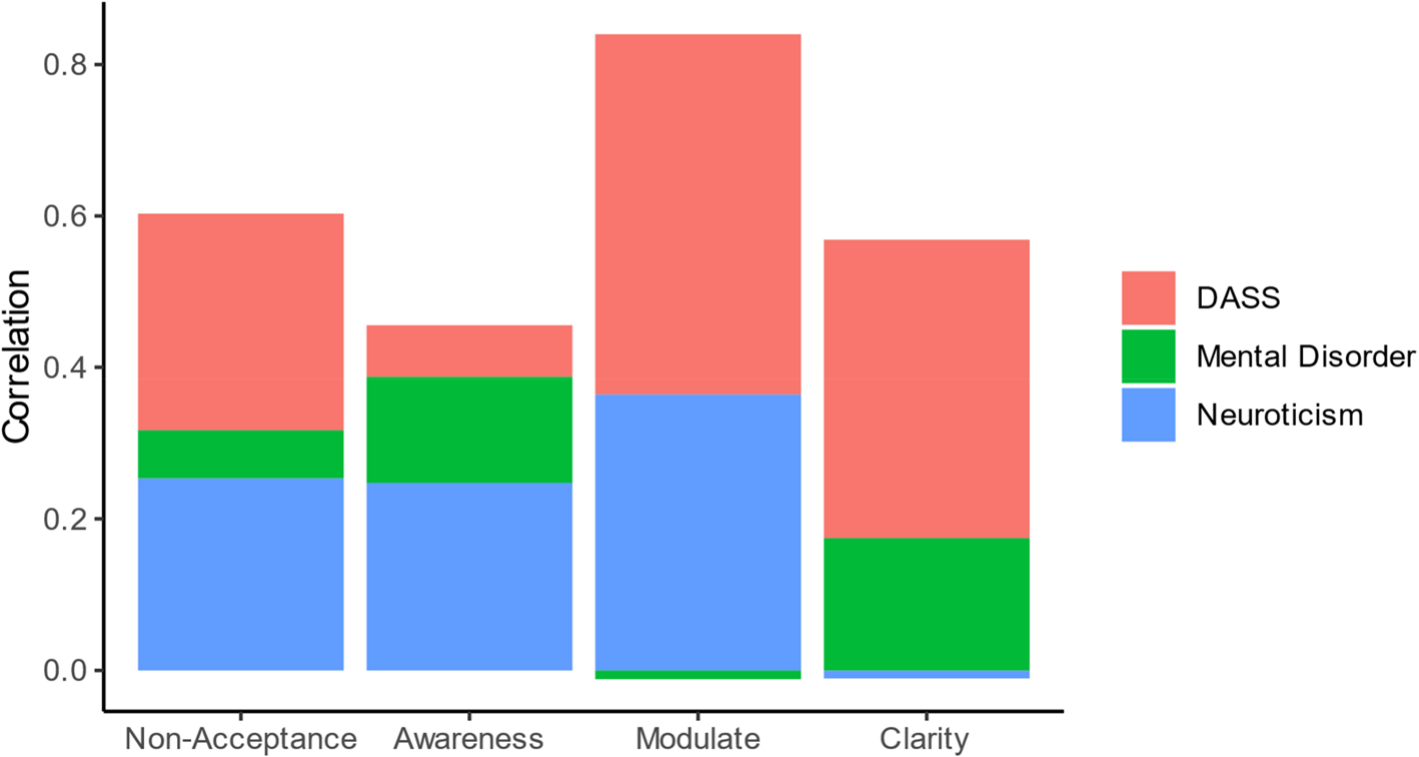
Latent correlations between S-DERS subscales and psychopathology-related outcomes

The fact that S-DERS subscales consist of a different number of items implies a differential weighting of these subscales when a global score is calculated (e.g., the Clarity scale has a lower weight than Non-Acceptance in a global S-DERS sum score as it only consists of two items). Therefore, we tested whether equally weighting all subscales increases correlations with psychopathology-related outcomes. We calculated mean scores instead of sum scores for each subscale. Then, we calculated a global scale score as the average over those four means, leading to an equal weight for all subscales independent of the number of items. Overall, we found that the previously used (implicit) differential weighting of scales in the S-DERS is superior to equally weighting all scales for the DASS (Δ*r* = 0.10), neuroticism (Δ*r* = 0.11), and having been diagnosed with a mental disorder (Δ*d* = 0.14). One possible explanation is that the overrepresented subscales (i.e., Non-Acceptance and Modulate) have stronger relations to the three outcomes, as indicated in Table 3. Still, this might also be a result of their higher reliability (i.e., equally representing Clarity in the total score lowers the overall reliability). Therefore, we also computed correlations between the latent (measurement error-free) S-DERS factors and the three outcomes within the ESEM framework (Fig. 3). This provided evidence for both explanations as latent effect sizes were much more similar than manifest effect sizes for the four subscales, but the Modulate scale still appeared to outperform the other measures in terms of cumulative effect sizes across all three outcomes. This was driven by strong relations to DASS and neuroticism.

## Discussion

We presented a reliable and valid German translation of the S-DERS together with insights from extended analysis on complex factor and network models, which might serve future studies on the temporal dynamics of emotion regulation difficulties in the laboratory or daily life.

As most previous studies, we started by assuming a common factor model, implying that latent causes (Non-Acceptance, Modulate, Awareness, and Clarity) influence the responses on the individual items. Here, we found that generally no overall factor of “emotion regulation difficulties” can be assumed, based on comparisons to a higher-order and bi-factor model. Hence, a global S-DERS score would be simply an amalgam of four correlated but distinct factors. While a S-DERS sum score might still be statistically useful (e.g. for prediction), this observation speaks against the existence of general state-based emotion regulation difficulties as a coherent psychological construct.

We further confirmed the observation from the trait DERS that comprehensive cross-loadings are necessary for sufficient model fit [17]. This indicates that specific self-reported behaviors are not a simple mono-causal consequence of clearly delineated sub-facets of emotion regulation difficulties (e.g., Non-Acceptance), but rather influenced by all sub-facets to some degree. Hence, there is considerable complexity in the relation between single indicators of emotion regulation difficulties and latent sub-facets. An ESEM model is only one of many possible highly complex models, which might capture the actual complexities in these relations.

Network models are an alternative to such common-cause factor models, which are difficult to compare on the basis of fit measures [12]. Recognizing one’s emotions appears as a prerequisite to acknowledging them at face-value, showing a potential causal interpretation of substantial cross-loadings for item 7. Similarly, we found that item 5 might serve as a connector between the Modulate and the Non-Acceptance scale. A hypothesis-generating causal interpretation would be, for example, that feeling very bad about oneself (item 5) may make someone expect increasingly depressed mood (item 15), which makes one feel overwhelmed (item 21) and believing one will continue to feel this way for a long time (item 10). Therefore, zooming in on the potential causal relations between individual difficulties might be an important target for future clinical research.

There are several limitations in our study. First, while our sample composition resembled that of the original S-DERS validation and had a substantial proportion of people with a history of diagnosed mental disorders, this is still a highly educated, mostly young, female sample, imposing constraints on generalizability. Secondly, while our mood induction did increase negative affect, there was no association between affective reactivity and the S-DERS. In turn, there was a substantial association between affect *before* but not after the mood induction and the S-DERS, despite the latter being administered *after* the mood induction. One potential explanation might be that despite the mood induction being successful, its only small-to-moderate effect size was not sufficient to observe an association with reactivity, as participants might fastly revert to their affective set point before the mood induction. Put simply, our mood induction might have been insufficiently strong to overwrite the emotion regulation difficulties participants experienced before the mood induction.

Thirdly, while the EFA models largely conformed to the criteria for conceptual replication of the original S-DERS study (with the exception of two slightly larger cross-loadings discussed above), only the SRMR index crossed the preregistered threshold for a stricter test of model fit. These thresholds were determined based on default standards in the field, which has been increasingly shown to have major shortcomings. Fit measures other than SRMR have been shown to lead to over-extraction of factors, underestimation of model fit for small sample sizes, and particularly bad diagnostic performance for determining the number for factors of RMSEA based on simulations [18, 20, 29]. For our sample size, more recent studies have shown that flexible cutoffs on the SRMR based on simulations have the best performance for model diagnostics [14, 23, 25, 30]. When performing such analyses with the FCO package [30], we found a cutoff of SRMR = 0.055, which our ESEM model passes. Generally, we believe that for DERS-like structures, psychometric decisions based on fixed absolute literature-based thresholds might not be a fruitful path. In items and scales with such complex interrelations, it is highly unlikely that a CFA simple structure without any cross-loadings or an ESEM structure with cross-loadings for all items can reflect the true data generating model. In the long run, such formal models of emotion regulation difficulties might be improved based on clinical theory and evidence targeting causal relations.

Lastly, while all items had sufficiently large loadings on their targeted factor, two items showed stronger cross-loadings than expected. First, item 17 of the Modulate factor “My emotions feel out of control” had a substantial cross-loading on the Clarity factor. In the original trait-DERS, the item wording was “I experience my emotions as overwhelming and out of control”, which might have benefitted a stronger alignment with the Modulate versus the Clarity factor. For example, if someone is confused about their feelings (item 14, Clarity), this might be closer to experiencing emotions as out of control than them being overwhelming. Still, given that we confirmed previous patterns of only moderate discriminant validity for the S-DERS subscales, it might be advantageous to retain this item translation for consistency with the English S-DERS. Also, the trait-DERS wording combines two different items into one, “out of control” and “overwhelming”, which are both already present in the trait-DERS as separate items. This might artificially improve factor solutions by effective doubling of content, which could even favor the removal of this item from all DERS versions entirely.The second item with substantial cross-loadings was item 7 of the Clarity scale (“I have no idea how I am feeling”), which also loaded on the Awareness scale. We would argue it is almost trivial that the awareness someone has of their current emotions influences whether they have an idea how they are feeling. In fact, a well-powered study on the trait-DERS found that the Awareness and Clarity scales were not distinct [1]. This highlights general questions on the causal structure of the (S-)DERS.

## Conclusion

Our study provides a robust German translation and validation of the S-DERS, expanding its applicability for research on state-like emotion regulation difficulties. Beyond validation, our findings challenge the assumption of a unidimensional construct of general emotion regulation difficulties, demonstrating that the four subscales—Non-Acceptance, Modulate, Awareness, and Clarity—are correlated but distinct. This has important theoretical and practical implications, suggesting that while a global S-DERS score may be statistically useful, it does not reflect a coherent psychological entity.

Moreover, our structural analyses reinforce findings from trait-based research [17] that emotion regulation difficulties cannot be cleanly separated into distinct latent causes, given the pervasive cross-loadings in CFA models. This supports the use of ESEM as a flexible modeling approach that accommodates the complexity of emotion regulation processes. However, as factor models inherently assume latent common causes, future work may also benefit from network-based perspectives. Our exploratory network analysis highlights potential causal relationships between individual items, offering a hypothesis-generating framework for future studies to investigate the dynamic interplay of emotion regulation difficulties in real-world settings.

Taken together, these findings underscore the importance of moving beyond static trait assessments to study the fluid nature of emotion regulation difficulties. Future research should further explore these dynamics beyond experimental settings, using intensive longitudinal designs in daily life, integrating both factor-analytic and network approaches to capture the full complexity of state-based emotion regulation.

## Supplementary Information

Supplementary Material 1.

## Data Availability

Preregistration, codebook, data, and analysis code can be found at: https://osf.io/ebj6u/.

## Abbreviations

S-DERS: State Difficulties in Emotion Regulation Scale
DASS-21: Depression, Anxiety, and Stress Scale
BIS-15: Barrett Impulsivity Scale
AAQ-II: Acceptance and Action Questionnaire II
FFMQ: Five Facet Mindfulness Questionnaire (short form)
