# Supplementary material for: Translation, validation and extended factor models of the German State Difficulties in Emotion Regulation Scale (S-DERS)
Source: Borderline Personal Disord Emot Dysregul. 2025 Jul 1;12:25. doi: 10.1186/s40479-025-00299-y (PMC12211531; doi:10.1186/s40479-025-00299-y)
Supplement: Supplementary file 1 — Supplementary Material 1. [file 40479_2025_299_MOESM1_ESM.docx]

**A German version of the State Difficulties in Emotion Regulation Scale (S-DERS): Translation, Validation and
Extended Factor Models**

***-Supplements-***

Sicorello, M., Elsaesser, M., Kolar, D.

**S-DERS (State Difficulties in Emotion Regulation Scale)**

Bitte lesen Sie jede Aussage und geben Sie an, wie sehr diese auf IHRE GEFÜHLE IN DIESEM MOMENT zutrifft.

|  | In diesem Moment… | Überhaupt nicht | Etwas | Teils, teils | Sehr | Völlig |
| --- | --- | --- | --- | --- | --- | --- |
| 1. | Ich fühle mich schuldig dafür, dass ich mich so fühle. | 1 | 2 | 3 | 4 | 5 |
| 2. | Ich achte gerade darauf, wie ich mich fühle. | 1 | 2 | 3 | 4 | 5 |
| 3. | Ich fühle mich außer Kontrolle. | 1 | 2 | 3 | 4 | 5 |
| 4. | Es ist mir peinlich, dass ich mich so fühle. | 1 | 2 | 3 | 4 | 5 |
| 5. | Ich denke gerade sehr schlecht über mich. | 1 | 2 | 3 | 4 | 5 |
| 6. | Ich erkenne gerade meine Gefühle an. | 1 | 2 | 3 | 4 | 5 |
| 7. | Ich habe keine Ahnung, wie ich mich gerade fühle | 1 | 2 | 3 | 4 | 5 |
| 8. | Ich schäme mich dafür, dass ich mich so fühle. | 1 | 2 | 3 | 4 | 5 |
| 9. | Ich habe gerade Schwierigkeiten, die Dinge zu tun, die ich genau jetzt tun müsste. | 1 | 2 | 3 | 4 | 5 |
| 10. | Ich glaube, dass ich mich noch für eine lange Zeit weiter so fühlen werde. | 1 | 2 | 3 | 4 | 5 |
| 11. | Mir liegt etwas an meinen jetzigen Gefühlen. | 1 | 2 | 3 | 4 | 5 |
| 12. | Ich ärgere mich über mich selbst, dass ich mich so fühle. | 1 | 2 | 3 | 4 | 5 |
| 13. | Ich habe gerade Schwierigkeiten, mein Verhalten zu kontrollieren. | 1 | 2 | 3 | 4 | 5 |
| 14. | Ich bin verwirrt darüber, wie ich mich fühle. | 1 | 2 | 3 | 4 | 5 |
| 15. | Ich glaube, dass ich mich am Ende sehr niedergeschlagen fühlen werde. | 1 | 2 | 3 | 4 | 5 |
| 16. | Ich nehme mir gerade Zeit herauszufinden, was ich gerade wirklich fühle. | 1 | 2 | 3 | 4 | 5 |
| 17. | Meine Gefühle erlebe ich als außer Kontrolle. | 1 | 2 | 3 | 4 | 5 |
| 18. | Ich bin von mir selbst genervt, weil ich mich so fühle. | 1 | 2 | 3 | 4 | 5 |
| 19. | Ich glaube, dass meine Gefühle berechtigt und wichtig sind. | 1 | 2 | 3 | 4 | 5 |
| 20. | Ich habe das Gefühl, schwach zu sein, weil ich mich so fühle. | 1 | 2 | 3 | 4 | 5 |
| 21. | Meine Gefühle fühlen sich überwältigend an. | 1 | 2 | 3 | 4 | 5 |

**Auswertung S-DERS**

Gesamtskala = SUMME (Item1-21)

Subskala Non-Acceptance = SUMME(Item8+Item4+Item1+Item5+Item12+Item20+Item18)

Subskala Modulate = SUMME(Item13+Item17+Item10+Item3+Item15+Item21+Item9)

Subskala Awareness = SUMME(Item6+Item11+Item2+Item19+Item16)

Subskala Clarity = SUMME(Item14+Item7)

| **Table S1** |
| --- |
| *S-DERS Items by Scale* |
| **Non-Acceptance** |
| Ich schäme mich dafür, dass ich mich so fühle. 8 |
| Es ist mir peinlich, dass ich mich so fühle. 4 |
| Ich fühle mich schuldig dafür, dass ich mich so fühle. 1 |
| Ich denke gerade sehr schlecht über mich. 5 |
| Ich ärgere mich über mich selbst, dass ich mich so fühle. 12 |
| Ich habe das Gefühl, schwach zu sein, weil ich mich so fühle. 20 |
| Ich bin von mir selbst genervt, weil ich mich so fühle. 18 |
| **Modulate** |
| Ich habe gerade Schwierigkeiten, mein Verhalten zu kontrollieren. 13 |
| Meine Gefühle erlebe ich als außer Kontrolle. 17 |
| Ich glaube, dass ich mich noch für eine lange Zeit weiter so fühlen werde. 10 |
| Ich fühle mich außer Kontrolle. 3 |
| Ich glaube, dass ich mich am Ende sehr niedergeschlagen fühlen werde. 15 |
| Meine Gefühle fühlen sich überwältigend an. 21 |
| Ich habe gerade Schwierigkeiten, die Dinge zu tun, die ich genau jetzt tun müsste. 9 |
| **Awareness** |
| Ich erkenne gerade meine Gefühle an. 6 |
| Mir liegt etwas an meinen jetzigen Gefühlen. 11 |
| Ich achte gerade darauf, wie ich mich fühle. 2 |
| Ich glaube, dass meine Gefühle berechtigt und wichtig sind. 19 |
| Ich nehme mir gerade Zeit herauszufinden, was ich gerade wirklich fühle. 16 |
| **Clarity** |
| Ich bin verwirrt darüber, wie ich mich fühle. 14 |
| Ich habe keine Ahnung, wie ich mich gerade fühle. 7 |

| **Table S2** | | |
| --- | --- | --- |
|  | **Original Items (English)** | **German Translation** |
| 1. | I feel guilty for feeling this way. | Ich fühle mich schuldig dafür, dass ich mich so fühle. |
| 2. | I am paying attention to how I feel. | Ich achte gerade darauf, wie ich mich fühle. |
| 3. | I feel out of control. | Ich fühle mich außer Kontrolle. |
| 4. | I am embarrassed for feeling this way. | Es ist mir peinlich, dass ich mich so fühle. |
| 5. | I am feeling very bad about myself. | Ich denke gerade sehr schlecht über mich. |
| 6. | I am acknowledging my emotions. | Ich erkenne gerade meine Gefühle an. |
| 7. | I have no idea how I am feeling. | Ich habe keine Ahnung, wie ich mich gerade fühle |
| 8. | I feel ashamed with myself for feeling this way. | Ich schäme mich dafür, dass ich mich so fühle. |
| 9. | I am having difficulty doing the things I need to do right now. | Ich habe gerade Schwierigkeiten, die Dinge zu tun, die ich genau jetzt tun müsste. |
| 10. | I believe that I will continue feeling this way for a long time. | Ich glaube, dass ich mich noch für eine lange Zeit weiter so fühlen werde. |
| 11. | I care about what I am feeling. | Mir liegt etwas an meinen jetzigen Gefühlen. |
| 12. | I am angry with myself for feeling this way. | Ich ärgere mich über mich selbst, dass ich mich so fühle. |
| 13. | I am having difficulty controlling my behaviors. | Ich habe gerade Schwierigkeiten, mein Verhalten zu kontrollieren. |
| 14. | I am confused about how I feel. | Ich bin verwirrt darüber, wie ich mich fühle. |
| 15. | I believe that I am going to end up feeling very depressed. | Ich glaube, dass ich mich am Ende sehr niedergeschlagen fühlen werde. |
| 16. | I am taking time to figure out what I am really feeling. | Ich nehme mir gerade Zeit herauszufinden, was ich gerade wirklich fühle. |
| 17. | My emotions feel out of control. | Meine Gefühle erlebe ich als außer Kontrolle. |
| 18. | I am irritated with myself for feeling this way. | Ich bin von mir selbst genervt, weil ich mich so fühle. |
| 19. | I believe that my feelings are valid and important. | Ich glaube, dass meine Gefühle berechtigt und wichtig sind. |
| 20. | I feel like I’m a weak person for feeling this way. | Ich habe das Gefühl, schwach zu sein, weil ich mich so fühle. |
| 21. | My emotions feel overwhelming. | Meine Gefühle fühlen sich überwältigend an. |
